# Rare variant association studies: considerations, challenges and opportunities

**DOI:** 10.1186/s13073-015-0138-2

**Published:** 2015-02-23

**Authors:** Paul L Auer, Guillaume Lettre

**Affiliations:** School of Public Health, University of Wisconsin-Milwaukee, Milwaukee, WI 53201-0413 USA; Montreal Heart Institute and Université de Montréal, Montreal, Quebec H1T 1C8 Canada

## Abstract

Genome-wide association studies (GWASs) have successfully uncovered thousands of robust associations between common variants and complex traits and diseases. Despite these successes, much of the heritability of these traits remains unexplained. Because low-frequency and rare variants are not tagged by conventional genome-wide genotyping arrays, they may represent an important and understudied component of complex trait genetics. In contrast to common variant GWASs, there are many different types of study designs, assays and analytic techniques that can be utilized for rare variant association studies (RVASs). In this review, we briefly present the different technologies available to identify rare genetic variants, including novel exome arrays. We also compare the different study designs for RVASs and argue that the best design will likely be phenotype-dependent. We discuss the main analytical issues relevant to RVASs, including the different statistical methods that can be used to test genetic associations with rare variants and the various bioinformatic approaches to predicting *in silico* biological functions for variants. Finally, we describe recent rare variant association findings, highlighting the unexpected conclusion that most rare variants have modest-to-small effect sizes on phenotypic variation. This observation has major implications for our understanding of the genetic architecture of complex traits in the context of the unexplained heritability challenge.

## Introduction

Simple (or Mendelian) diseases such as sickle cell anemia and cystic fibrosis are caused by mutations in single genes. These mutations, which may have variable penetrance and expressivity, are causal. On the other hand, complex (or common) human diseases such as myocardial infarction or schizophrenia result from the combined effect of multiple genetic variants and environmental stresses. Genetic variants associated with complex human diseases do not cause diseases but rather influence the risk of developing them. The focus of this review is on the role of rare genetic variants in complex human diseases. Genome-wide association studies (GWASs) have successfully uncovered thousands of robust associations between common variants and complex traits and diseases. As GWAS-based consortia have expanded to include hundreds of thousands of samples [1,2], the role of common variation in the genetics of complex traits is becoming well-characterized. However, the genetic markers evaluated in a GWAS typically do not represent rare genetic variation, defined in this review as variants with a minor allele frequency (MAF) <1%. To evaluate comprehensively the contribution of rare genetic variants to complex traits and diseases, DNA sequencing-based approaches and custom arrays have recently been developed and deployed on a large scale [3-5]. In addition, there are many different types of study designs and analytic techniques that have been developed specifically to maximize the power of rare variant association studies (RVASs). With these tools, investigators have begun describing the contribution of rare variants on complex traits and diseases.

The original excitement over RVASs sparked from targeted gene sequencing experiments, which identified rare coding variants with strong effects on phenotypic variation. These included genetic variation in *ABCA1* and *PCSK9*, associated with high-density lipoprotein (HDL)- and low-density lipoprotein (LDL)-cholesterol levels, respectively [6-8]. In particular, the translational success of the identification of the *PCSK9* gene association [9,10] raised hopes that RVASs would yield a large number of strong effect variants useful in predictive/personalized medicine, and a plethora of new drug targets. After a few years of RVASs, our expectations have met reality: most rare variants identified to date have modest-to-weak effect sizes. Instead of considering this a glass half empty, we argue that RVASs still hold tremendous promise in biomedicine, and have significant potential for drug development. Here, we provide a brief overview of the various technologies that are useful for discovering and/or genotyping rare variants. We also discuss, using recent findings as examples, analytical issues that are relevant to RVASs, including which statistical methods to use to test genetic associations with rare variants, genotype imputation, replication challenges, and variant annotation strategies. Finally, we comment on how we envision RVASs may impact our understanding of the genetic architecture of complex traits.

## Identifying rare variants

RVASs are now possible because of next-generation DNA sequencing (NGS) technologies and the development of software to process, control the quality of, and call DNA variation from the vast amount of sequence reads generated [11]. The 1000 Genomes Project sequenced the whole genomes of >2,500 individuals from 26 populations around the world, and similar or even larger projects are underway [5,12,13]. Thus, it is now possible to collect rare genetic variants in large samples and test their role in human phenotypic variation, including in disease risk. However, comprehensive whole-genome sequencing (WGS) at high coverage (>20×) remains prohibitively expensive in large cohorts (see Table 1 for cost estimates based on specific study designs). WGS at low depth (<10×) is a less expensive alternative that can generate high-quality variant calls when combined with imputation methods (see below). The 1000 Genomes Project successfully employed this strategy [5,12]. Indeed, if the budget is limiting, there is more statistical power to find genetic associations when sequencing more individuals at lower depth than fewer samples at high coverage [14].

**Table 1:** **Comparison of strategies for rare variant association studies**

**Approach**	**Design**	**DNA target size**	**Technology**	**Cost/sample (US$)**
Whole-genome sequencing	2,000 individuals at 30× (high depth)	3.3 gigabases	For example, Illumina (DNA library and sequencing)	~4,000^a^
	2,000 individuals at 5× (low depth)	3.3 gigabases		~800
Whole-exome sequencing	2,000 individuals at 80×	50 to 70 megabases	Agilent SureSelect (capture); Illumina (DNA library and sequencing)	~750
Targeted sequencing of candidate genes	2,000 individuals at 100×	500 kilobases (exons from ~100 genes)	TruSeq Custom Amplicon Illumina (capture); Illumina (DNA library and sequencing)	~325
	2,000 individuals at 100×	100 kilobases (exons from ~20 genes)		~250
	5,000 individuals at 100×	100 kilobases (exons from ~20 genes)		~125
Exome array	10,000 individuals	~250,000 coding variants	Illumina ExomeChip array	~70

We provide cost estimates for next-generation DNA sequencing or genotyping experiments using different study designs.

^a^With the recently developed Illumina HiSeq X Ten platform, whole-genome sequencing at high coverage is 60 to 70% cheaper. We do not recommend or endorse any specific companies or products. Cost estimates do not include bioinformatics processing.

Because 98% of the human genome is non-coding and therefore more difficult to interpret, enrichment methods were developed to capture only a fraction of the genome before building sequencing libraries. Whole-exome sequencing (WES) relies on the solution-based capture of exons, and several companies (such as Illumina (San Diego, USA), Roche’s Nimblegen (Madison, USA) and Agilent’s SureSelect (Santa Clara, USA)) offer well-designed exome-wide capture reagents. In the last few years, WES has been instrumental in defining the culprit gene(s) for dozens of Mendelian diseases [15]. Recently, the National Heart, Lung, and Blood Institute (NHLBI) Exome Sequence Project (ESP) sequenced the exome of 6,515 individuals to characterize protein-coding variants and to identify rare genetic variations associated with different complex human diseases and traits [3,16].

In many applications - for instance, to follow up candidate genes from a GWAS or to screen clinically important genes in a genetic clinic - sequencing 50 to 1,000 kilobases is often desirable (Table 1). The first option for targeted NGS is to design and order custom solution-based capture reagents that target only the sequences of interest (for example, Agilent’s SureSelect or Roche’s Nimblegen offer these products). The second method, which is usually cheaper but may result in sequencing libraries of lower complexity, uses PCR to amplify the targeted sequences (for example, Illumina Truseq Amplicon, Haloplex, Raindance, Fluidigm) prior to NGS. Both enrichment methods have been successfully applied to find rare genetic variants [17-20].

Another approach to performing RVASs consists of genotyping rare variants directly. Large-scale projects like the 1000 Genomes Project and ESP have generated an extensive catalogue of coding DNA sequence variants. Once genomic coordinates and alleles are known (from sequencing), it is possible to interrogate these variants using standard genotyping arrays. After an important contribution from the human genetics community, exome-wide genotyping arrays (exome chips) are now available (for instance, those developed by Illumina and Affymetrix) that test hundreds of thousands of exonic variants at modest costs (Table 1). Genotyping data also have the advantage of being computationally simpler to analyze than NGS data. But exome chips do have important limitations. First, they are not as exhaustive as sequencing and will miss a large amount of very rare genetic variation. Second, because most of the sequence data used to design the arrays that have been developed so far were from Europeans or individuals of European ancestry, exome chips may not interrogate rare variants in other populations very well. Despite these limitations, exome chips have already been used successfully to identify rare coding variants associated with insulin traits [4], liver disease [21], lipid levels [22,23] and blood cell counts [24].

## Designs for rare variant association studies

Motivated by the prediction that rare variants have large effect sizes that explain some of the missing heritability in complex traits [25], a variety of study designs can be utilized for finding rare variant associations. The success of such a study depends on multiple factors that influence the range of observable effect sizes (for example, sample size and the magnitude and direction of natural selection) [26].

### Extreme phenotype sampling

For studies of quantitative traits, it has been shown that the power to detect rare variant associations can be increased by sampling from the extremes of the trait distribution [27,28]. To do so, typically the phenotype (or a transformed version of the phenotype) is assumed to follow a normal distribution. Then, the largest and smallest *n*^th^ percentile of the distribution are chosen for study, where *n* is typically less than five. For disease outcomes, the power of the study may be increased by sampling from the extremes of known risk factors (such as looking at early onset disease) [29]. For instance, Lange and colleagues [30] sampled from the extremes of the distribution of LDL-cholesterol levels to select individuals for WES. They combined these ‘extreme’ samples with other ‘normal’ samples to discover a burden of rare and low-frequency variants in the *PNPLA5* gene that were associated with LDL-cholesterol. Similarly, Emond and co-workers [31] chose samples for exome sequencing based on the extremes of the first time to *Pseudomonas* infection in individuals with cystic fibrosis. This approach yielded a novel genetic association between rare coding variants in the *DCTN4* gene and time to first *Pseudomonas* infection, a surrogate measure of cystic fibrosis severity. More recently, Flannick and colleagues [32] selected individuals from the extremes of type 2 diabetes (T2D) risk by including both young and lean T2D cases as well as elderly, non-obese controls. The initial analysis discovered a nonsense variant in *SLC30A8* that was strongly protective against T2D. Additional genotyping of over 44,000 cases and controls confirmed a 53% reduction in T2D risk for carriers of the nonsense variant.

Although extreme sampling may boost the statistical power of a study to detect associations, data analysis often requires sophisticated statistical techniques to remove sampling bias [33,34]. Furthermore, the results may be difficult to generalize to the underlying population from which the extremes were drawn. For rare variants, tens of thousands of samples may still be necessary in order to detect modest effects even for extreme trait designs [27].

### Population isolates

Owing to a variety of demographic forces (for example, famine, war, migration), many subpopulations around the world have undergone extreme population bottlenecks, and have become isolated and remained so for many generations [35,36]. These extreme bottlenecks and the resultant population isolates produce several genetic and phenotypic consequences that are interesting to a geneticist. From a phenotypic perspective, population isolates often demonstrate environmental and cultural homogeneity, resulting in a lack of phenotypic variability that can be advantageous for an association study. Furthermore, because of this reduced genetic diversity (due to the bottleneck) and increased genetic drift (due to isolation), population isolates often show a lack of concordance in allele frequencies with other non-isolated populations [37]. Because the power to detect an association is partly determined by allele frequency, population isolates can be very useful in discovering rare variant associations [37]. If the disease-causing variant(s) occurs at high frequency in the population isolate, the power to detect an association may be high.

Recently, a study of WGS data from 1,795 Icelanders identified a non-coding, low-frequency variant associated with prostate cancer [38]. The risk allele was observed at much higher frequency in Iceland (3% in cases, 1% in controls) compared to other populations that served for replication (for example, 0.4% and 0.1% in Spanish cases and controls, respectively). The same group later identified several low-frequency and rare variants that were associated with T2D [39]. These variants occurred at much higher frequency in Icelandic and Danish populations compared to an Iranian population used for replication. Perhaps the most well-known examples of risk variants found in a population isolate are the *BRCA1* and *BRCA2* mutations, which occur at high frequency in the Ashkenazi Jewish population and are associated with risk for breast and ovarian cancer [40]. However, the lack of genetic diversity in population isolates can serve as a serious disadvantage as well. Disease-causing variant(s) may be exceedingly rare, or monomorphic in the population isolate, leaving little chance of detecting an association.

### Family studies

A different type of design for identifying disease-causing rare variants is to study a family with multiple affected members. Often referred to as ‘family studies’, such a design involves sequencing co-affected family members and searching for overlapping variants that co-segregate with the condition of interest. Both linkage-based and genetic association methodologies are amenable to family studies. This type of design has been very successful in identifying large effect, highly penetrant mutations that underlie Mendelian disorders [41,42]. However, for many common diseases co-segregation analysis cannot sufficiently distinguish among a large set of candidate pathogenic variants [43]. Given the challenges of performing a comprehensive analysis of pedigree sequencing data, most studies rely on a series of *ad hoc* filtering criteria, although there has been recent progress in developing unified and rigorous methods for analyzing sequence data from pedigrees [44]. If the disease-causing variant occurs with high frequency in the affected families (compared with the general population), a family study may provide a significant boost in statistical power compared to other designs. For family studies, as well as population isolates, this is sometimes referred to as ‘hitting the jackpot’, because investigators are essentially hoping to ‘get lucky’ by observing the disease-causing variant with high frequency in the affected families (or population isolate) [45].

**Figure 1:**
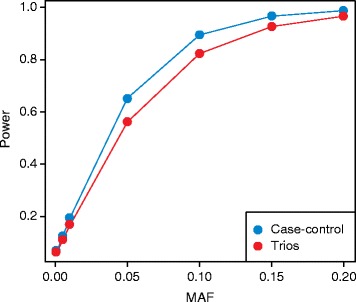
**Comparison of power for trios and case–control designs.** Power to detect associations for 10,000 cases and 10,000 controls (blue) and 10,000 trios (red) across a range of minor allele frequencies (MAFs). Power was calculated with a significance threshold of *P* < 0.05, a prevalence of 0.1 and a relative risk of 1.1, using the Genetic Power Calculator tool [112].

In addition to co-segregation analysis, genotype data from trios (an affected offspring and his or her parents) are often used in studies with a family-based design. The transmission disequilibrium test (TDT) [46] has been developed to detect associations in these types of designs. For rare diseases (for instance, those with a prevalence <0.5%) the TDT for *n* number of trios provides the same statistical power as a case–control design, with *n* cases and *n* controls [47]. For common diseases, case–control designs are more powerful (Figure 1).

The underlying genetic architecture of the trait of interest determines which study design is best powered to detect the association [48]. For most complex traits, the genetic architecture is unknown or at best partially known. Thus, there is no way to predict *a priori* which design will be most powerful. Both population-based studies (such as extreme phenotype sampling or population isolates) and family designs are powerful and useful in differing contexts and should serve as complementary, rather than competing, approaches for uncovering the genetic contribution of rare variants to complex traits.

## Statistical methods for RVASs

In the GWAS literature, single nucleotide polymorphisms (SNPs) that reach association *P*-values <5 × 10^−8^ are generally labeled as significant genome-wide. The HapMap Project established this significance threshold by examining common genetic variation in populations of European, African and Asian ancestry [49]. The researchers on the project noted that when considering linkage disequilibrium (LD), there are approximately one million independent loci in the human genome (*α* = 5 × 10^−8^ is the Bonferonni-corrected threshold for a million tests). However, because rare variants are more numerous and less correlated with each other than common variants, a simple *α* = 5 × 10^−8^ threshold is not enough to declare significance in association studies that target rare variants. Thus, RVASs suffer from an increased multiple testing burden and a decrease in statistical power owing to the rarity of individuals carrying these variant alleles. It is generally recognized that a better strategy for analyzing rare variants is to combine them within units of association, defined using gene annotations, genomic coordinates or functional characterization (see below). Because rare variants are traditionally grouped by genes, these tests are referred to as gene-based tests and use *α* = 2.5 × 10^−6^ as a significance threshold in a genome-wide search (assuming approximately 20,000 genes in the human genome).

We can divide most gene-based tests into two main categories: burden and variance-component tests [50]. In its simplest form, a burden test asks whether individuals that carry a rare variant in a gene are phenotypically similar to individuals that do not. To run this analysis, one simply aggregates carriers of rare variants and compares their phenotype mean or disease prevalence (for quantitative or dichotomous traits, respectively) with non-carriers. The generalization of this approach in various software packages (CAST [51], CMC [52], VT [53], and so on) allows for the inclusion of covariates when appropriate, or for different weighting of each variant based on allele frequency or other functional annotations. The main limitation of burden tests is that they assume that all tested variants influence the phenotype in the same direction. However, we know from several examples in animal models (such as *CED9* alleles in *Caenorhabditis elegans* and their effect on programmed cell death) and Mendelian genetics (such as loss- and gain-of-function *PCSK9* alleles and their effect on LDL-cholesterol) that the same gene can carry alleles that have dramatic phenotypic consequences, but in opposite directions. To allow for this scenario, tests that consider the distribution of genetic effects (that is, the variance instead of the mean) for a group of variants were developed. For instance, the widely used SKAT test offers flexibility in terms of covariate adjustment, study design (for example, modeling family structure) and different variant prioritization/weighting strategies [54]. Recently, Lee and colleagues [55] combined burden and variance-component tests of rare variants into the SKAT-O test, which maximizes discovery power under different genetic architecture models. The development of new methods to analyze rare genetic variants in association studies, as well as to control for confounders such as population stratification, is a very active research area in statistical genetics (recently reviewed in [56]). In particular, recent work suggested that approaches that are commonly used in GWASs to account for population structure, such as principal component analysis and linear mixed effect models, are in many instances not appropriate for RVASs [57-60]. Indeed, because stratification of rare variants is different and often stronger than for common variants, inflated significance of gene-based tests is possible despite traditional correction methods. Considering family-based designs or including spatial/geographical information in the tests might be strategies to limit confounding due to population structure [57].

As for GWASs of common variants, large sample sizes are required to identify significant associations in RVASs. Because ethical concerns usually prevent the sharing of genotype and phenotype information between collaborators, genetic consortia tend to analyze summary statistic data; that is, association results for each variant across all participating studies [61]. This approach works well when we analyze one variant at a time, but is limited when considering groups of correlated genetic markers aggregated in the same test. The solution is to generate a matrix that summarizes the correlation (LD) between each marker. Investigators can then share association results from each variant as well as the LD matrix, such that the correlation between markers is considered when meta-analyzing test statistics. The LD matrix offers the additional advantage of allowing for conditional analyses - testing the independence of an association signal when several markers are genotyped at the same locus - without the need to access genotype data. This meta-analysis strategy is implemented in software packages such as rareMETAL [62] and skatMeta [63] (see also [64]).

## Genotype imputation

Owing to the costs associated with genotyping or sequencing large numbers of samples, many studies of rare variant associations do not have sufficiently large sample sizes for a well-powered analysis. Genotype imputation can be used to increase sample sizes by imputation of sequence variants (from a sequencing study) into large numbers of samples with genome-wide array data. Genotype imputation (or *in silico* genotyping) is a statistical technique for predicting genotypes at variants that are not directly measured [65], and several methods are available for large-scale imputation of genotypes [66-69]. Genotype imputation utilizes a set of reference samples that have been densely genotyped to identify segments of haplotypes that are shared with the study or ‘target’ population. Data from the HapMap and the 1000 Genomes projects serve as popular reference sets for imputation [5,70].

Genotype imputation is particularly convenient for large-scale GWAS meta-analyses and has largely been utilized in this context. Studies that contribute to meta-analyses often do not genotype their data on the same platform. Thus, imputed genotypes are frequently used to ‘square-off’ datasets so that the meta-analysis can consider the union (rather than the intersection) of the variants that were considered by each study [71,72].

In addition to GWAS meta-analyses, genotype imputation can be used to search for rare variants associated with complex traits. Auer and co-workers took sequence data from the ESP and imputed them into over 13,000 samples with genome-wide array data [3,73]. This approach yielded associations between variants at the *LCT* locus and circulating white-blood-cell counts as well as between variants in the *MPL* gene and platelet counts. Imputation of ESP data was also used to find loci associated with height [74]. By sequencing the whole genomes of 2,630 Icelanders, followed by imputation into large sets of GWAS data, novel associations between rare variants in *APP* and Alzheimer's disease and between rare variants in *PDX1* and T2D were identified [39,75]. The SardiNIA Medical Sequencing Discovery Project imputed WGS data from 828 unrelated subjects to find loci associated with immune-cell levels [76], and the genomes of 1,325 individuals from the Minnesota Twin Family registry were sequenced and imputed into approximately 7,000 subjects to find loci that were associated with a variety of psychophysiological endophenotypes [77]. These studies highlight the power of utilizing study-specific reference panels for imputation.

Study-specific reference panels can also enhance the quality of imputation for rare variants. Duan and colleagues [78] showed that imputation with the ESP reference panel outperformed imputation with the 1000 Genomes reference panel for rare coding variants. Furthermore, imputation of variants from a Sardinian-specific reference panel into Sardinian GWAS samples provided improved imputation accuracy compared with 1000 Genomes imputation [79]. This study showed that a combination of 1000 Genomes data and a Sardinian reference panel provided the best imputation quality for rare variants, compared to using either reference panel alone.

Imputation of sequence variants from related individuals or extended pedigrees can also boost imputation accuracy. The Genome of the Netherlands project sequenced 769 Dutch samples at 14× coverage, derived from 231 trios and 19 quartets, resulting in 998 unrelated haplotypes. Imputation of these variants into other Dutch as well as English and Italian samples showed improvement over 1000 Genomes imputation [80], highlighting how imputation accuracy can be improved by matching the ancestries of the reference and target panels. In addition, the trio design enabled phasing that accurately reconstructed long-range haplotypes, leading to improved imputation of rare variants [81].

Imputation accuracy decreases with MAF, making it difficult to impute very rare variants. However, imputation accuracy also increases with the size of the reference set. With larger reference sets, imputation of very rare variants should become more accurate. To this end, there has been a recent effort to establish a Haplotype Reference Consortium that contains sequence data on over 30,000 subjects [82]. With these data as a reference set, accurate imputation of variants with MAFs as low as 0.01% may be possible.

A gold-standard association study would include directly measured genotypes from tens or even hundreds of thousands of study samples. However, even though the cost of sequencing continues to fall, it is not currently feasible directly to genotype rare variants across the genome in tens of thousands of samples. Thus, for the foreseeable future, genotype imputation is likely to remain an efficient and cost-effective means of inferring genotypes and increasing sample sizes for association studies.

## Functional annotation of rare variants

In addition to the choice of statistical approach to test for rare variant associations, another important consideration is whether one should include all discovered rare variants in a group-based analysis. Many tools and resources exist to annotate DNA sequence variants (Table 2). For instance, upon sequencing a candidate gene, should you include all coding as well as non-coding (promoter, untranslated regions, introns) variants in the test? Even for coding variants, should synonymous changes (DNA changes that result in the same encoded amino acids because of redundancy in the genetic code) be considered? Variants that are probably detrimental - nonsense, splice site and frameshift - are often prioritized because of their enrichment among true loss-of-function alleles, such as in human syndrome genes [83-85]. For most missense variants, however, the distinction between phenotypically active and neutral alleles is not straightforward, especially as population genetic theory and empirical observations suggest that even functional missense variants will mostly have small effect sizes [86]. Large efforts are currently underway to attempt to catalogue all loss-of-function alleles in the human genome [87].

**Table 2:** **Partial list of tools and resources to annotate DNA sequence variants**

**Tool/resource**	**Description**	**URL**	**Reference**
CADD	A framework that integrates multiple annotations into one metric by contrasting variants that survived natural selection with simulated mutations	http://cadd.gs.washington.edu/	[88]
ENCODE	Annotation of potential functional elements (for example, histone tail modifications) in several cell lines	https://www.encodeproject.org/	[89]
Epigenomics Roadmap	Annotation of potential functional elements (for example, DNAse I hypersensitive sites) in many human tissues and primary cells	http://www.roadmapepigenomics.org/	[90]
FANTOM5	Annotation of transcriptional enhancers in many human tissues and primary cells through detection of bidirectional capped transcription	http://enhancer.binf.ku.dk/	[91]
GERP	Identifies constrained elements in multiple alignments by quantifying substitution deficits	http://mendel.stanford.edu/SidowLab/downloads/gerp/	[92]
HaploReg	Visualization of DNA polymorphisms along with their predicted chromatin state, their sequence conservation across mammals, and their effect on regulatory motifs	http://www.broadinstitute.org/mammals/haploreg/haploreg.php	[93]
Phen-Gen	Method that combines patients' disease symptoms and sequencing data with prior domain knowledge to identify the causative genes for rare disorders	http://phen-gen.org/about.html	[94]
PolyPhen-2	A tool that predicts the possible impact of an amino acid substitution on the structure and function of a human protein using straightforward physical and comparative considerations	http://genetics.bwh.harvard.edu/pph2/	[95]
RegulomeDB	A database that annotates SNPs with known and predicted regulatory elements in the intergenic regions of the human genome using gene expression, ENCODE and literature-mining data	http://regulomedb.org/	[96]
RVIS	This score is designed to rank genes in terms of whether they have more or less common functional genetic variation relative to the genome-wide expectation given the amount of apparently neutral variation the gene has	http://chgv.org/GenicIntolerance/	[97]
SIFT	Predicts whether an amino acid substitution affects protein function based on the degree of conservation of amino acid residues in sequence alignments derived from closely related sequences	http://sift.jcvi.org/	[98]
VEP	Determines the effect of your variants (SNPs, insertions, deletions, CNVs or structural variants) on genes, transcripts and protein sequence, as well as regulatory regions.	http://useast.ensembl.org/info/docs/tools/vep/index.html?redirect=no	[99]

CNV, copy number variant; SNP, single nucleotide polymorphism.

Bioinformatic methods based on conservation, structural information and/or amino acid physico-chemical properties have been developed to estimate the likelihood that a given missense variant is detrimental [100,101]. But these algorithms are not perfect, as recently illustrated by the study of genetic coding variation in the T2D gene *PPARG* [102]. In that study, investigators sequenced *PPARG* in approximately 20,000 individuals and identified 49 new rare non-synonymous variants. In aggregate, these rare variants were not associated with T2D risk, even when considering in the analysis their frequency or *in silico* predictions. The authors then tested the effect of each rare *PPARG* non-synonymous variant in an adipocyte differentiation assay, and noted that only 12 of these variants were truly functional. Interestingly, individuals that carry these rare functional *PPARG* alleles have a sevenfold increase in their odds of developing T2D [102]. This example is a striking demonstration that simply relying on computer-based predictions to assess the impact of rare missense variants is not ideal, and emphasizes a need to develop high-throughput functional characterization methods.

**Figure 2:**
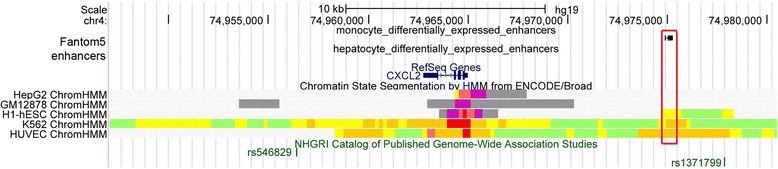
**Functional annotation of regulatory sequences in the human genome.** Genome tracks from the UCSC Genome Browser. *CXCL2* (blue) encodes a chemokine produced by activated monocytes and neutrophils at sites of inflammation. Single nucleotide polymorphisms (SNPs; rs546829 and rs1371799, green) are associated with monocyte count by a genome-wide association study. The red box upstream of *CXCL2* includes a predicted enhancer identified in monocytes by FANTOM5 (black rectangles). FANTOM5 did not annotate an enhancer in hepatocytes, a less relevant cell type for *CXCL2*. Using histone tail modification information, ENCODE predicted strong enhancers (orange) at the same position in erythroleukemic (K562) and endothelial (HUVEC) cells. Chr, chromosome; hESC, human embryonic stem cell; HMM, hidden Markov model; kb, kilobases.

Non-coding variants are even more difficult to ascertain, and until recently the estimation of their biological impact relied mostly on conservation scores. Publically available data generated by the ENCODE, Roadmap Epigenomics and FANTOM5 projects now offer an alternative strategy to focus on ‘more likely’ functional non-coding variants in genetic association studies [89-91]. These projects used applications of NGS (for example, chromatin immunoprecipitation assays, DNase I hypersensitive site mapping and cap analysis of gene expression) to localize DNA sequences that may regulate gene expression, such as promoters and enhancers in different human cells and tissues (Figure 2). These annotated regulatory regions are enriched for GWAS findings, and the enrichment is usually stronger when the relevant cells or tissues are considered (for instance, SNPs associated with multiple sclerosis preferentially localized in regulatory sequences defined in immune cells) [90,103-106]. As for SNPs, we can use this functional annotation of the human genome in RVASs by grouping variants not only by genes but also on the basis of regulatory sequence coordinates defined in the appropriate human cell type or tissue.

## Strategies for replication

An important consequence of GWASs has been the standardization of the statistical evidence needed for an association to be accepted. An independent replication of results and a genome-wide cutoff of *P* < 5 × 10^−8^ are now considered requirements for novel associations to be published [107]. For publication of novel rare variant associations, independent replication is equally important, but can be more challenging than for SNPs identified by GWASs. Indeed, because these variants are rare and often population-specific, it might be difficult to find appropriate replication panels. For instance, Sanna and colleagues [108] reported a rare *LDLR* variant (MAF = 0.5%) associated with LDL-cholesterol that is polymorphic only in Sardinians. In such cases, attempting to replicate the association by discovering other variants in the same gene that are associated with the same phenotype in other populations might be the only approach to confirm the initial genetic association.

Rare variant associations are typically tested in two ways: by considering each variant individually (similar to in a standard GWAS analysis); or by aggregating rare variants into a single unit (typically a gene) and utilizing any number of aggregate rare variant association tests [52-55]. Thus, rare variant association signals are observed in two different varieties, either by implicating a single variant or by implicating an entire gene. Replication of a rare variant association can therefore also be thought of in two ways, by either replicating the association with a single variant or with an entire gene.

Replication of single variant associations can be done in a multitude of ways. Using an independent population (that is, one that is distinct from the population used for discovery), it is possible to perform selected genotyping of the variant of interest, selected genotyping of a perfect tag SNP, or *in silico* genotyping (that is, imputation) of the selected variant, although the latter option is suboptimal. If the association signal from the independent population is statistically significant, then the discovery has been replicated. Note that the proper threshold for statistical significance for replication is *P* < 0.05/*n*, where 0.05 is the traditional significance threshold for a single test and *n* is the total number of variants brought forward for replication; this corresponds to the Bonferroni correction for multiple testing.

There are several different options for replication of aggregate or ‘gene-based’ associations. Liu and Leal [109] considered various replication strategies in this context and compared the power of each approach. Briefly, using independent samples, researchers can choose to sequence the gene that was implicated in the discovery analysis, or genotype the same set of variants that were observed in the gene in the discovery analysis. The difference between the two approaches being that by sequencing the gene it is possible to observe variants in the replication stage that were not observed in the discovery stage. All other things being equal (for example, error rates and cost), Liu and Leal [109] show that sequencing in the replication stage is consistently more powerful than genotyping across a number of different scenarios. However, sample size in the replication stage is the most important determinant of power. Practically speaking, whichever strategy (sequencing or genotyping) results in the largest sample size should be the preferred approach.

Both strategies for replication have been successful in practice. Crosby and co-workers [110] describe how they identified an association between rare variants in the *APOC3* gene and triglyceride levels using exome sequencing in 3,734 subjects. For replication of this finding, four loss-of-function variants in *APOC3* were included on the Illumina ExomeChip and successfully genotyped in 41,671 additional subjects. The gene-based test replicated at *P* = 7 × 10^−6^. In contrast, Emond and co-workers [31] discovered an association with rare variants in the *DCTN4* gene and time to *Pseudomonas* infection in individuals with cystic fibrosis. This discovery was made via exome sequencing and was replicated by Sanger sequencing of the entire gene.

## Rare variants in common human diseases

With the technology and analytic tools now in place, and motivated by successes such as the identification of low-frequency coding variants in *PCSK9*, which led to the development of new therapies to treat hypercholesterolemia [7-9], RVASs are starting to characterize (and quantify) the contribution of rare variants to human phenotypic variation. Although there are a few exceptions (for example, the association of *PCSK9* with LDL-cholesterol and coronary artery diseases, *TREM2* and *APP* with Alzheimer’s disease), our early findings suggest that most rare variants will have small effect sizes on phenotypes, and may therefore have only limited value in predictive medicine. But that should not undermine the intrinsic value of RVASs. For one, such experiments may lead to the identification of new genes implicated in human diseases. For instance, using the ExomeChip approach, we identified a series of rare missense variants in the chemokine receptor gene *CXCR2* that are associated in aggregate with low white-blood-cell counts in individuals of European ancestry [24]. *CXCR2* had not been implicated in white-blood-cell biology by GWASs. We also confirmed that rare familial mutations in *CXCR2* cause congenital neutropenia through an effect on leukocyte migration [24].

RVASs of coding variants are also useful to explore GWAS loci further. Indeed, one of the limitations of GWAS findings is that they usually highlight non-coding SNPs in LD with many other markers across large genomic intervals that may contain several genes. Although we generally accept that most GWAS SNPs have regulatory functions [90], it is sometimes difficult to localize the causal genes based only on this information. Although it is not a definitive proof, finding coding variants that are associated with the same trait is a strong argument in favor of specific gene(s) within GWAS loci being causal. As such, RVASs and GWASs should not be considered as competitive but rather as complementary approaches to study common diseases and complex traits. As an example, two groups recently identified a low-frequency missense variant in *TM6SF2* that is associated with total cholesterol levels and alanine transaminase, and that explains GWAS signals at the locus for coronary artery and non-alcoholic fatty liver diseases [21,23]. We anticipate that one of the main outcomes of RVASs will be to clarify mechanistically - through the identification of causal genes and coding loss- and gain-of-function alleles - results from GWASs.

## Conclusions and future directions

New technological advances now enable human geneticists to explore the contribution of low-frequency and rare genetic variants in phenotypic variation. For obvious reasons, the scientific community initially focused on the 2% of the human genome that is protein coding, identifying new genes implicated in common human diseases and complex traits, or new alleles located in previously implicated loci (either by GWASs or Mendelian genetics). While we continue to explore coding variation, we need to start considering how to tackle the remaining 98% of the human genome. Indeed, there is no reason why rare non-coding variants should not influence human phenotypic variation. However, there remain challenges ahead in terms of developing statistical methods and experimental tools to distinguish rare neutral variants from functional variants. Without doubt, some of the progress in this field will come from studying other populations (isolates or different ethnic groups) or using different designs (such as family-based designs) where rare variants might be more common. We also need to develop phenotype-relevant high-throughput assays in cells or model organisms to characterize the biological impacts of these rare variants. Despite these challenges, we believe that studying the influence of rare genetic variants in human biology is a worthwhile endeavor. The premise of RVASs was that rare genetic variants would have strong effect sizes on phenotypes; with a few exceptions, this is clearly not what we observe. However, we should not forget that we do not need to find strong-effect alleles to gain insights into human biology, disease pathophysiology, or to consider and develop new therapeutic strategies [111].

## Abbreviations

chr: Chromosome
CNV: Copy number variant
ESP: Exome Sequence Project
GWAS: Genome-wide association study
hESC: Human embryonic stem cell
HDL: High-density lipoprotein
kb: Kilobases
LD: Linkage disequilibrium
LDL: Low-density lipoprotein
MAF: Minor allele frequency
NGS: Next-generation DNA sequencing
NHLBI: National Heart, Lung, and Blood Institute
RVAS: Rare variant association study
SNP: Single nucleotide polymorphism
T2D: Type 2 diabetes
TDT: Transmission disequilibrium test
WES: Whole-exome sequencing
WGS: Whole-genome sequencing
